# BIM-integrated life cycle assessment of decentralized cement-based waste recycling in renovation projects

**DOI:** 10.1038/s41598-025-23173-x

**Published:** 2025-12-01

**Authors:** Ammar Younes, Emad Elbeltagi, Ahmad Ehab, Aboelkasim Diab, Mohammed Hamdallah, Amgad Abazeed

**Affiliations:** 1 Construction and Building Engineering Department, Faculty of Engineering, Arab Academy for Science, Technology and Maritime Transport (AASTMT), Aswan, 81511 Egypt; 2 Department of Civil Engineering, College of Engineering, Qassim University, Buraydah, 51452 Saudi Arabia; 3 Civil Engineering Department, Structural Program, Badr University in Cairo (BUC), Cairo, Egypt; 4 Department of Civil Engineering, Aswan University, Aswan, 81542 Egypt; 5 School of Engineering and Natural Resources, Civil and Environmental Engineering, University of Iceland IS, Reykjavík, Iceland; 6 Department of Integrated Geodesy and Cartography, AGH University of Krakow, al. A. Mickiewicza 30, Krakow, 30-059 Poland

**Keywords:** Concrete waste, Cement-based waste, Recycled concrete aggregates, BIM, Sustainability, Civil engineering, Engineering

## Abstract

Decentralized community recycling offers a sustainable pathway for managing 100% cement-based waste (CBW) from renovation activities, providing a viable alternative to centralized recycling facilities and landfilling. This study employs an integrated BIM-Life Cycle Assessment (LCA) framework to evaluate the use of recycled concrete aggregate as a substitute for natural aggregates in non-structural concrete. The CBW was dismantled, segregated, crushed, and graded to produce RCA. Thirty-six specimens were prepared in three mixes: CBW0 (100% NA), CBW1 (100% coarse RCA + 50% fine RCA, Styrene butadiene rubber/Water (SBR/W) = 1:25), and CBW2 (100% coarse RCA + 50% fine RCA, SBR/W = 1:50). A cradle-to-site LCA (A1-A4), following the EN 15,804 + A2 (adapted) baseline, quantified the environmental impacts of 1 m^3^ of concrete. Relative to conventional concrete (CC), CBW2 achieved the target for grade 20 concrete, reaching 16 MPa after 28 days while reducing overall environmental impacts by 29%. Phase-specific savings reached 23.4% (A1-A3) and 75% (A4). These outcomes demonstrate the potential of decentralized recycling and BIM-LCA integration to balance mechanical performance with environmental efficiency in sustainable construction.

**Supplementary Information:**

The online version contains supplementary material available at 10.1038/s41598-025-23173-x.

## Introduction

The construction sector is a significant contributor to global environmental impacts, accounting for approximately 36% of total energy consumption and 39% of GHGs across the globe^1^. Among construction materials, concrete is the most extensively used, ranking second only to water in terms of overall human consumption^2^. Annual global concrete production is estimated at 30 billion metric tons^3^, driving the demand for approximately 4.3 billion tons of cement and 19.4 billion tons of aggregates^4^. In parallel, construction, renovation, and demolition activities generate nearly 10 billion metric tons of waste globally, with an estimated 35% ending up in landfills^5^. Within this context, CDW refers to the debris produced throughout the lifecycle of built assets, including during the construction, refurbishment, and deconstruction of buildings, bridges, and other infrastructure^6^. Across the world, CW constitutes the largest share of CDW; in the United States, Australia, and China, it accounts for approximately 85%, 81%, and 45%, respectively^7^. In Egypt, CDW is predominantly composed of dense materials such as concrete, bricks, sand, mortar, and ceramic tiles, with concrete alone representing nearly half of the total waste mass^8^. Mitigating the environmental impacts—particularly CO_2_ emissions—associated with the cement and GC industry has emerged as a major focus of global research efforts^9^. GC is defined by the incorporation of recycled materials and/or the implementation of environmentally responsible manufacturing practices, while maintaining high performance throughout the building’s life cycle^10^. Among its various properties, compressive strength is considered the most critical performance metric, influenced by mix proportions, cement type, and aggregate characteristics. The diversity and availability of sustainable GC constituents can enhance mechanical properties, optimize material performance, and contribute to environmentally responsible construction practices^11^. CW has become increasingly prevalent globally, with considered a valuable resource for recycling and reusing as aggregates^12^. Consequently, the usage of CW, such as RCA, in the A1 phase and the production of RAC in the A3 phase can deliver substantial environmental benefits^13^. While RCA can come from multiple sources, the majority are recovered from CDW due to the strong interest in repurposing them, as highlighted in this study. Other potential sources include waste generated by concrete production plants^14^. Research suggests that recycling one ton of CW can yield roughly 650 kg of CRCA, 330 kg of FRCA, and 20 kg of residual waste^15^. Despite these benefits, the incorporation of high volumes of both CRCA and FRCA into RAC presents specific challenges that warrant further investigation. Increasing the substitution of NA with RCA typically reduces concrete workability, with a more pronounced decrease in slump compared to CC^16^. However, findings from one study indicate that substituting 100% of CRCA and 50% of FRCA in the repair of concrete elements can achieve compressive strengths comparable to those of CC^17^. Other research has demonstrated that using up to 60% CRCA and 30% FRCA, along with a water-to-cement (W/C) ratio of 0.48, can maintain acceptable concrete properties^18^. A 50% replacement ratio of NA with RCA is considered feasible for various concrete applications^19^. When both CRCA and FRCA reach a 100% replacement rate, a notable reduction in cube compressive strength occurs, ranging from 36% to 42%^20^. A growing trend involves using materials derived from recovered demolition debris in Non-structural concrete (NSC) applications^21^. NSC, defined as concrete with a compressive strength of less than 25 MPa^22^, is commonly used in concrete driveways, sidewalks, curbs, and ornamental urban features, among others^23^. A study on NSC reported that replacing 75% of NA with RCA resulted in a 23% increase in splitting tensile strength, while the compressive strength reached 18.3 MPa at a cement content of 300 kg/m^3^^24^.

Alongside material innovations, BIM plays a critical role in promoting sustainable construction by enabling resource optimization, enhancing energy efficiency, and minimizing waste through various simulations and analyses^25^. Several strategies have been proposed to integrate BIM with life cycle assessment (LCA), facilitating the adoption of BIM in environmental assessment. These strategies have been systematically reviewed and categorized, yet they largely overlook decentralized recycling logistics^26^. For instance, Wang et al. developed a conceptual framework combining LCA and BIM to assess the environmental impacts of CDW management under cradle-to-gate scenarios with centralized recycling plants; however, their work did not extend to cradle-to-site recycling^27^. Similarly, Soust-Verdaguer et al. reviewed BIM-LCA integration approaches from previous case studies, classifying methods according to data input, analysis, and output, while Obrecht et al. further organized integration approaches into three levels of automation: manual, semi-automated, and fully automated^28,29^. While these studies advance the understanding of integration typologies, they primarily focus on classification rather than addressing the interoperability and dynamic data-exchange challenges that arise during actual LCA implementation. To address this gap, the present study emphasizes a semi-automated BIM-LCA integration approach tailored to incorporate decentralized recycling logistics within cradle-to-site scenarios.

The motivation for this research centers on exploring decentralized community recycling as a sustainable alternative to centralized recycling plants, specifically for processing 100% CBW from building renovations through local district government units. This approach not only reduces transportation impacts but also enhances resource circularity at the community level. Further investigation is needed to assess the feasibility of combining CRCA and FRCA at higher replacement ratios, as their concurrent use has shown the potential to achieve performance characteristics comparable to CC, particularly with optimized mix designs. Such practices also provide substantial environmental benefits, especially when assessed through cradle-to-site LCA relative to CC production. In addition, integrating BIM with LCA can automate calculations, yielding more accurate and efficient sustainability evaluations.

Accordingly, this research aims to develop a decision support framework for assessing both material properties and environmental impacts of using CBW as RCA in NSC elements. Three mix scenarios were studied: one control mix (CBW0) and two recycled mixes (CBW1, CBW2). The recycled mixes incorporated 100% CRCA and 50% FRCA substitution, with Styrene butadiene rubber (SBR) latex solution diluted at ratios 1:25 and 1:50 in order, while maintaining constant cement across all three mixes. A cradle-to-site LCA was conducted for each mix, covering stages A1-A4, and integrated with BIM to enable semi-automated assessment. The resulting comparative LCA-BIM framework was applied to a real-life construction project, demonstrating significant potential for environmental improvement through decentralized recycling and optimized RCA use.

## Research methodology

**Fig. 1:**
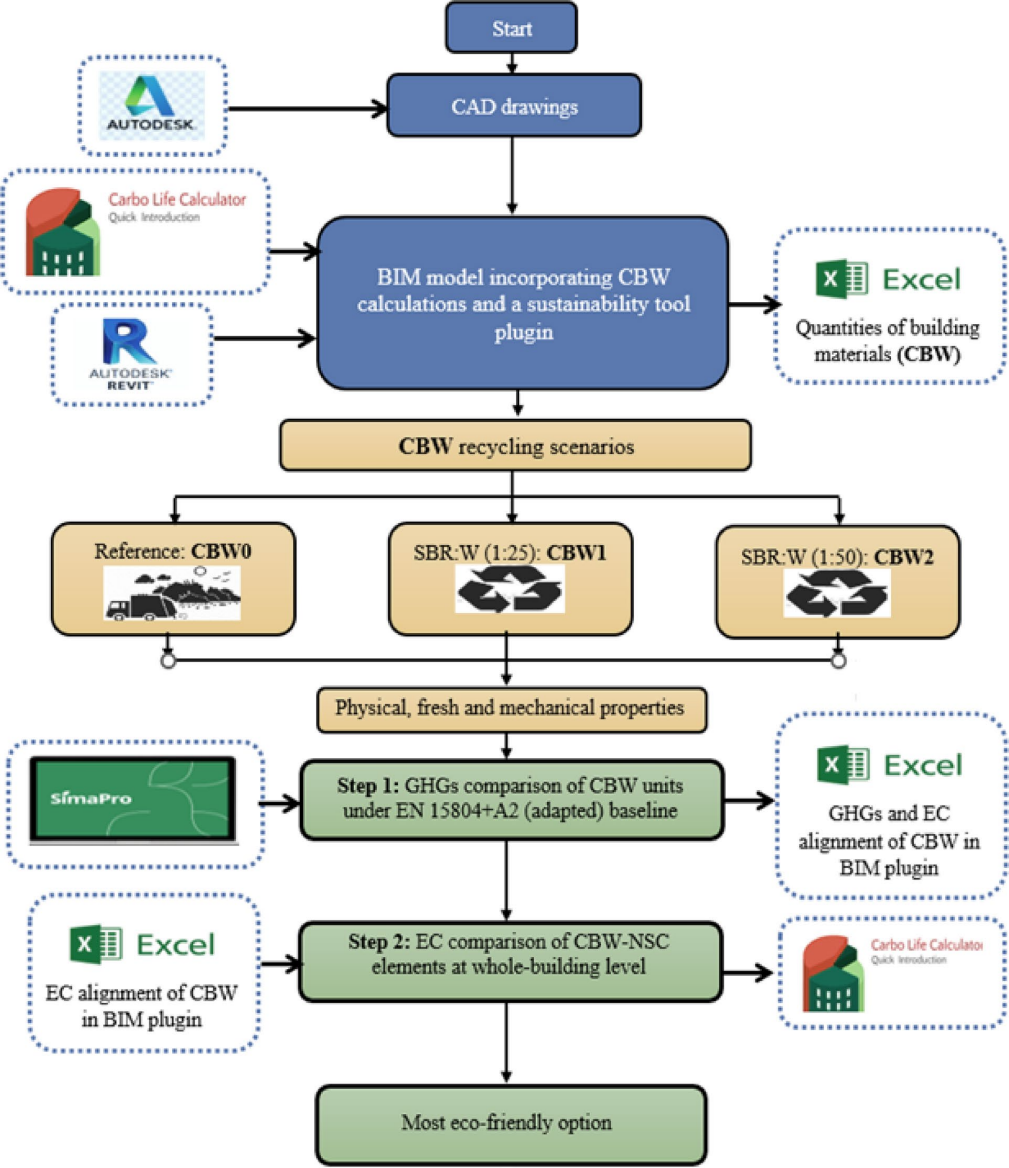
Flowchart of a semi-automated BIM-LCA process.

The proposed decision support system for CW, focusing on CBW, is intended for application in projects involving complete or selective disassembly, renovation, and rehabilitation, with a particular emphasis on CBW. The methodology for developing this system is presented in Fig. 1and consists of five main stages^1^: quantifying CBW through 3D modelling^2^; performing physical, fresh-state, and mechanical property analyses^3^; carrying out attributional LCA for recycling scenarios^4^; estimating the associated environmental savings; and^5^ supporting decision-making based on the scenario with lowest LCA impact. This framework is further complemented by a comprehensive environmental impact assessment and the detailed execution of a case study.

### BIM-LCA in CDW management

The built environment is rapidly advancing toward digitalization, with computer-aided technologies such as BIM at the forefront of this transformation. BIM plays a pivotal role in shaping the foundations for smart low-carbon cities^30^. As a collaborative platform, it enables architects, engineers, contractors, and facility managers to integrate their work seamlessly through a shared digital model within a Common Data Environment (CDE)^31^. In addition, BIM is increasingly applied to CDW estimation, as its models facilitate fast and reliable extraction of project-specific data^32^. BIM models provide detailed data on energy consumption and life cycle carbon emissions^33^.

The application of BIM in environmental burdens assessment enables the extraction of valuable life cycle inventory (LCI) data, including material specifications, quantity take-offs, and detailed information on building components. Moreover, integrating BIM models with LCA tools facilitates the automated retrieval and input of relevant data, thereby reducing manual effort and enhancing efficiency^34^. Wang et al. developed a BIM-based model to quantify carbon footprint from demolition waste^35^, while Jalaei et al. applied BIM to assess materials-to-waste conversion. However, this study focused on waste generation causes but overlooked impacts from waste processing^36^.

The integration process in BIM-LCA generally involves three fundamental steps: collecting data from BIM models and LCA databases, mapping corresponding elements, and executing data exchange via the chosen interoperability method. This workflow enhances data consistency and scalability, which are difficult to achieve using manual methods^37^. Moreover, the level of details (LOD) of a BIM model (from 100 to 500) directly affects the richness of geometric and material information, thereby improving the accuracy of environmental assessments^38^. Compared with conventional manual LCA, the use of a semi-automated BIM-LCA tool such as Carbo Life Calculator (Version 1.7.6, 7/9/2024. This is the first major update for Revit 2022 up to 2025., https://apps.autodesk.com/RVT/en/Detail/Index? id=5134144936403749174&appLang=en&os=Win64) demonstrated clear advantages: modelling time was reduced from 729 to 62 min (a 91.5% efficiency gain)^39,40^, while maintaining high accuracy, with results showing 98.6% agreement with traditional LCA outcomes^41^. These findings confirm that BIM-LCA not only accelerates the process but also ensures reliable and consistent results.

### Case study: aswan city (Egypt)

**Fig. 2:**
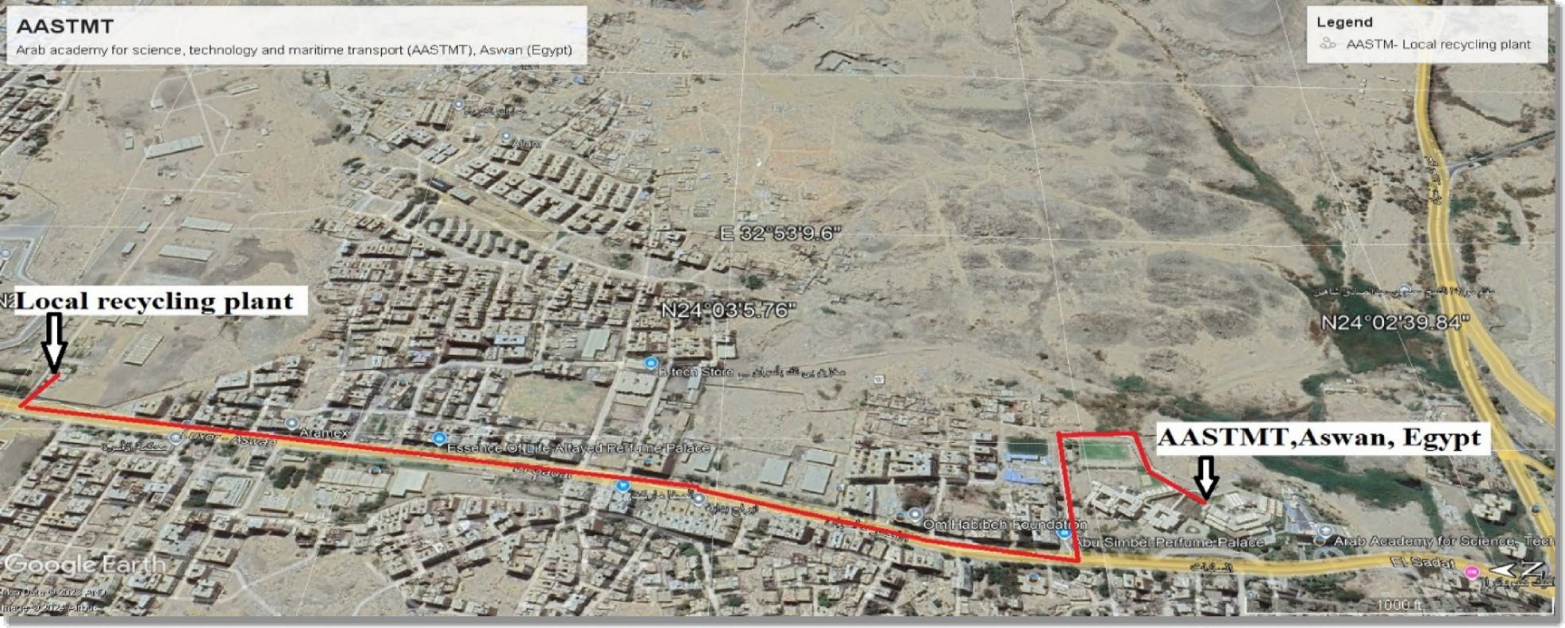
The local government recycling plant with a building under renovation, Aswan, Egypt (24° 3′ 8″ N 32° 53′ 0″ E). Imagery source: Google Earth Pro 7.3, Airbus 2024. https://www.google.com/earth/about/versions/#earth-pro.

**Fig. 3:**
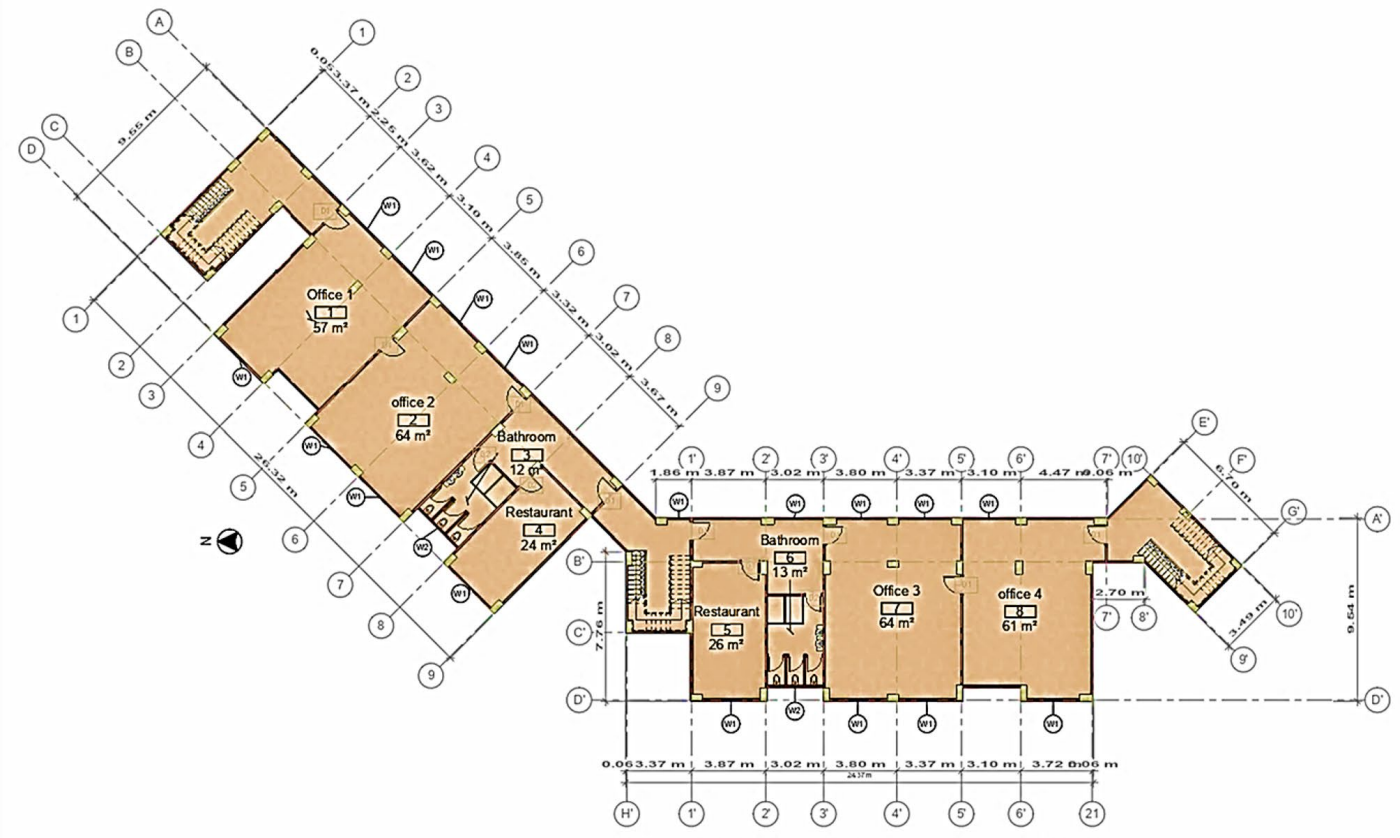
CAD-based plan of the case study.

Aswan city, located in southern Egypt, has an estimated population of 371,432 and covers an area of approximately 375 km^2^. The wider Aswan Governorate spans 62,726 km^2^ and hosts about 1.69 million inhabitants^42^. It is renowned as an international tourist destination, with its buildings and infrastructure undergoing frequent renovations, almost annually. Since most of the CDW, including CW, are sent to landfills and lack rehabilitation systems, this research aims to address this issue by using local government sites in every district by constructing a recycling concrete plant in this location, used to produce NSC applications for sale or use in government buildings. The current study adopted a multi-stepped approach in which the first step involved the acquisition of CAD (AutoCAD 2024, Autodesk Inc., https://www.autodesk.com/) drawings of the chosen case study. The Arab Academy for Science, Technology, and Maritime Transport (AASTMT), Aswan (Egypt), education building was considered for the case study. This education building was chosen as a case study due to the availability of its documents. Details regarding the building are given in Table 1 (refer to Fig. 2 and Fig. 3).

**Table 1:** Details of the AASTMT Building (case study).

Building properties and distances	Facts and figures
Gross internal floor area	432.25 m^2^
Number of floors	1
Location	Aswan (Egypt)
Raw-material transport (A2), NA, and cement	120.0 Km
Local recycling plant (A2) for RCA	5.0 Km
Centralized recycling plant	20.0 Km
Reverse logistics (A4) for concrete mix	5.0 Km

**Fig. 4:**
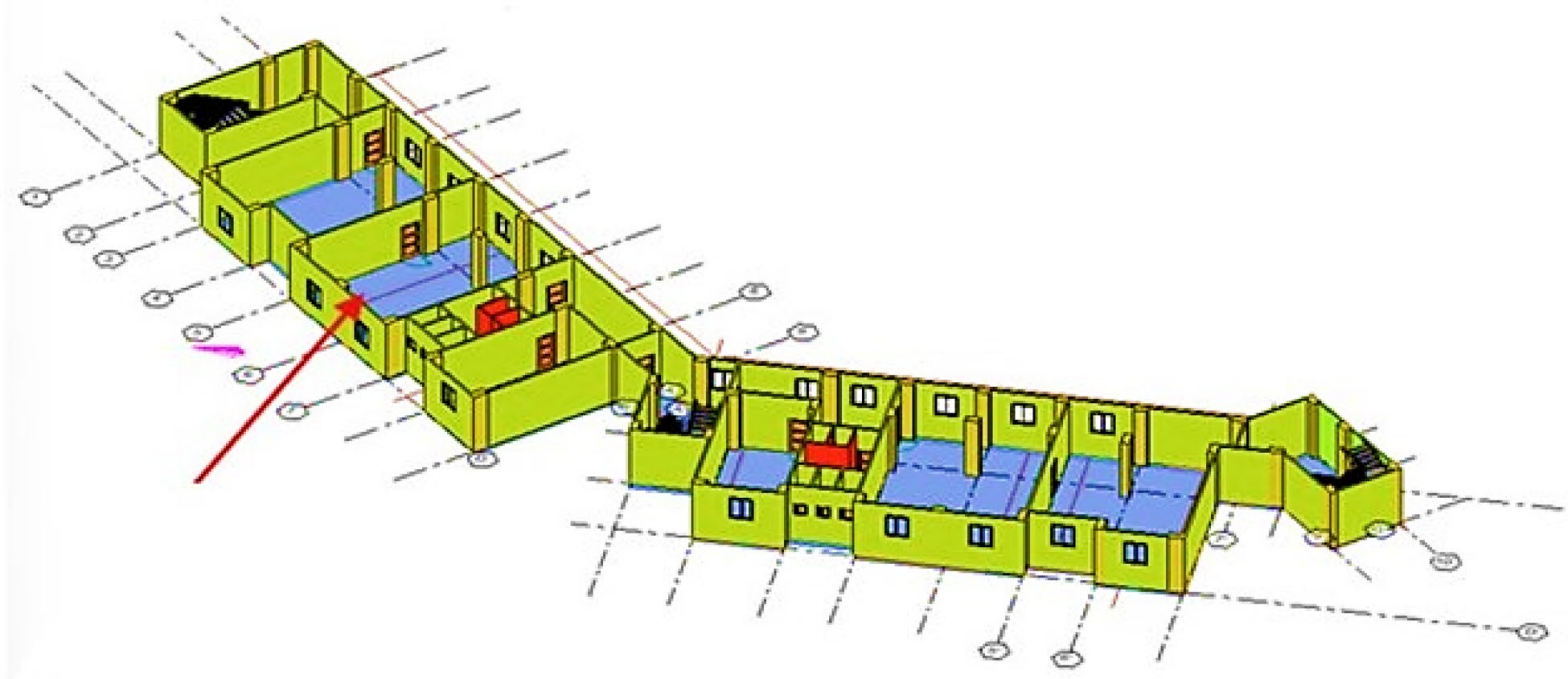
3D visualization of the case study.

In this study, CAD drawings were used to create a coordinated BIM model, including an architectural model developed based on construction drawings specifications, as shown in Figures and Fig. 4. The model was developed in Revit 2025 (Autodesk Revit 2025, Autodesk Inc., https://www.autodesk.com/products/revit/overview) of LOD 500 to perform functions such as quantity take-off and environmental analysis. The second part investigated recycling CBW generated from the renovation of the cement floor of the case study and performed an experimental program based on substitution of RCA instead of NA by adding SBR for the recycled mixes. The third part is to create an impact category excel sheet (Microsoft Excel (Microsoft Corporation, Microsoft 365 version, https://www.microsoft.com/microsoft-365/excel) (CSV) for each mix in the LCA tool and link with the BIM model as a plugin for automated calculations.

### Experimental program

The experimental program aims to examine the suitability of replacing both CNA and FNA with CRCA and FRCA, respectively. The investigation substituted NA with 100% CRCA and 50% FRCA, concurrently. Moreover, SBR, locally known as ADD 65, was introduced to the water in varying proportions (1:25 and 1:50). RCA and NA physical properties are investigated, including bulk density, specific gravity, water absorption, FM, clay and silt content, and PSD.

Consequently, fresh concrete workability was measured following the European Standard En 12350–2^43^. Thereafter, the mechanical characteristics were examined for thirty-six samples as follows; eighteen cubic specimens (15 × 15 × 15 cm) for compressive strength tests following standard 39 of the ASTM, using eighteen cylindrical specimens (15 cm in diameter × 30 cm in length) for indirect tensile strength tests consistent with ASTM C496/C496M guidelines^44^. To verify the reliability of the results, three replicates for each mixture were tested.

#### Materials for samples

In this study, crushed CNA and CRCA, both within the 4.75–9.5 mm size range, were employed. The FNA sourced from gravel and sand quarries, along with FRCA obtained from RCA passing the 4.75 mm sieve. The RCA materials (CRCA and FRCA) were derived from crushed CBW generated in the case study renovation project and mechanically processed (Fig. 2). Aggregate characterization was conducted following ASTM C136/C136M^45^. Ordinary Portland cement (CEM I 42.5 N) was used in compliance with the Egyptian Standard Specifications ESS 4756-1/2009^46^. Additionally, ADD 65 (SBR) was incorporated into the mortar at SBR/water (W) ratios of 1:25 and 1:50 to enhance bond strength. ADD 65 is a latex dispersion admixture containing SBR, designed to upgrade the performance of cementitious composites, and complies with ASTM C1059^47^.

#### Concrete mix design and testing procedures

All mixtures were designed to achieve a target compressive strength of 20 MPa at 28 days, as presented in Table 2. The mix design followed ACI 211.1 guidelines and employed the absolute volume method to determine constituent proportions^48^. In this study, “RCA” refers to both FRCA and CRCA, whereas “NA” denotes CNA and FNA. Specimens were tested at curing ages of 7 and 28 days to evaluate mechanical performance^49^. Three concrete mixes were prepared, producing a total of thirty-six specimens (Table 3). The reference mix (CBW0), composed entirely of NA, was used as a benchmark for recycling scenarios. Mixes CBW1 and CBW2 incorporated 100% CRCA and 50% FRCA, with SBR/W ratios of 1/25 and 1/50, respectively.

**Fig. 5:**
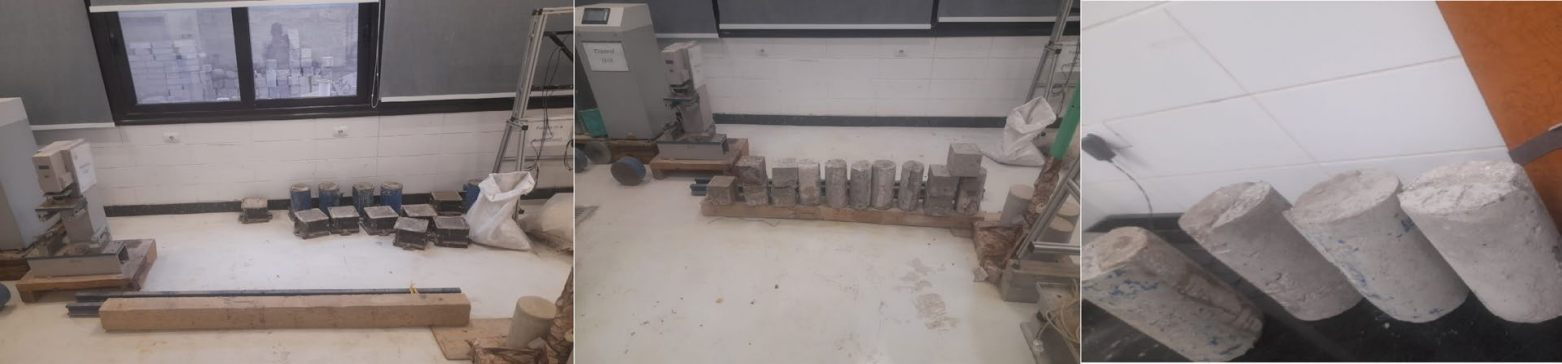
Preparation and casting of samples for testing.

All mixtures maintained a constant cement of 250 kg/m^3^ and a fixed coarse-to-fine aggregate ratio 5:3 as shown in Fig. 5. For comparison, the minimum cement contents recommended for NSC are 240, 280, and 320 kg/m^3^, depending on exposure conditions^50^.

**Table 2:** Mix design proportions for absolute volume method.

Component	Value
Design strength	20 MPa at 28 days
Cement content	250 kg/m^3^
Maximum aggregate size	9.5 mm
Coarse: fine	5: 3
SD	3.0 MPa (Very good control) ACI
Margin	1.64 × 3.0 = 4.92 MPa
Target mean strength	20 + 4.92 = 24.92 MPa

**Table 3:** Variations in concrete mixtures with RCA for 1 m^3^ of CBW.

Mixes	RCA%	Cement (kg)	W/C	SBR/W	CNA (kg)	FNA (kg)	CRCA (kg)	FRCA (kg)
CBW0 [reference]	0%	250	0.45	-	1263.0	758.0	-	-
CBW1	100% CRCA and 50% FRCA	250	0.60	1:25	-	379.0	1263.0	379.0
CBW2	100% CRCA and 50% FRCA	250	0.60	1:50	-	379.0	1263.0	379.0

### Environmental program

#### Comparative LCA: objectives, system boundary, and impact analysis

**Fig. 6:**
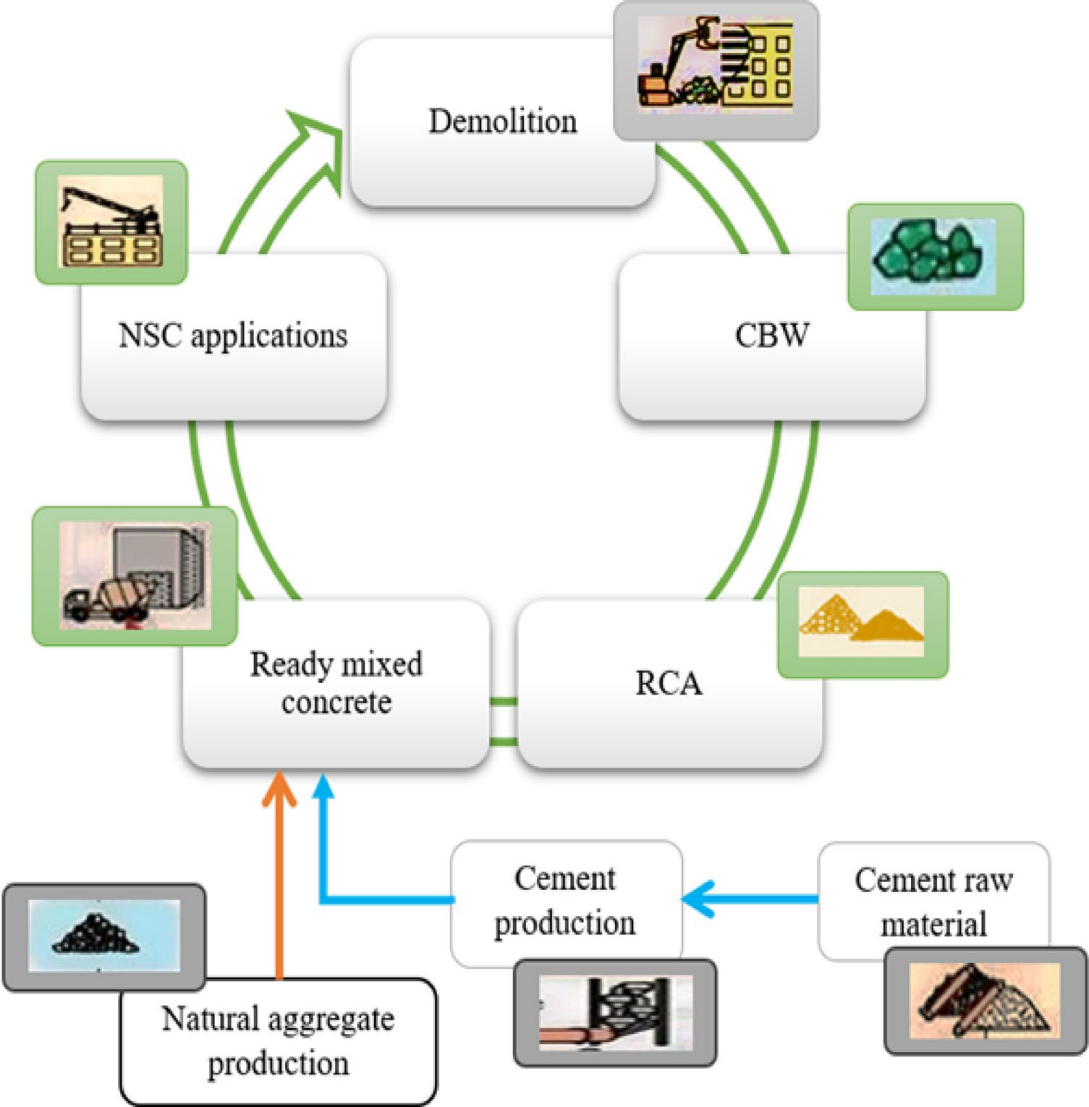
Schematic life cycle of RCA circularity.

Inefficient handling of CW can lead to severe environmental consequences, including global warming, ozone layer depletion, and aquatic eutrophication^51^. This study was conducted within the framework of LCA, following the principles of ISO 14040:2006^52^ and ISO14044:2006^53^, with adaptations from EN 15,804 + A2^54^. The assessment was structured around four core phases^1^: goal and scope definition^2^, LCI^3^, LCIA, and^4^ interpretation. This LCA analysis aims to assess the environmental effects of substituting RCA with NA obtained from CBW waste in RAC production impacts the environment as shown in Fig. 6. The study involves conducting a cradle-to-site LCA comparison across various concrete mixtures, including the “reference” CBW0, as well as mixtures CBW1 and CBW2.

**Fig. 7:**
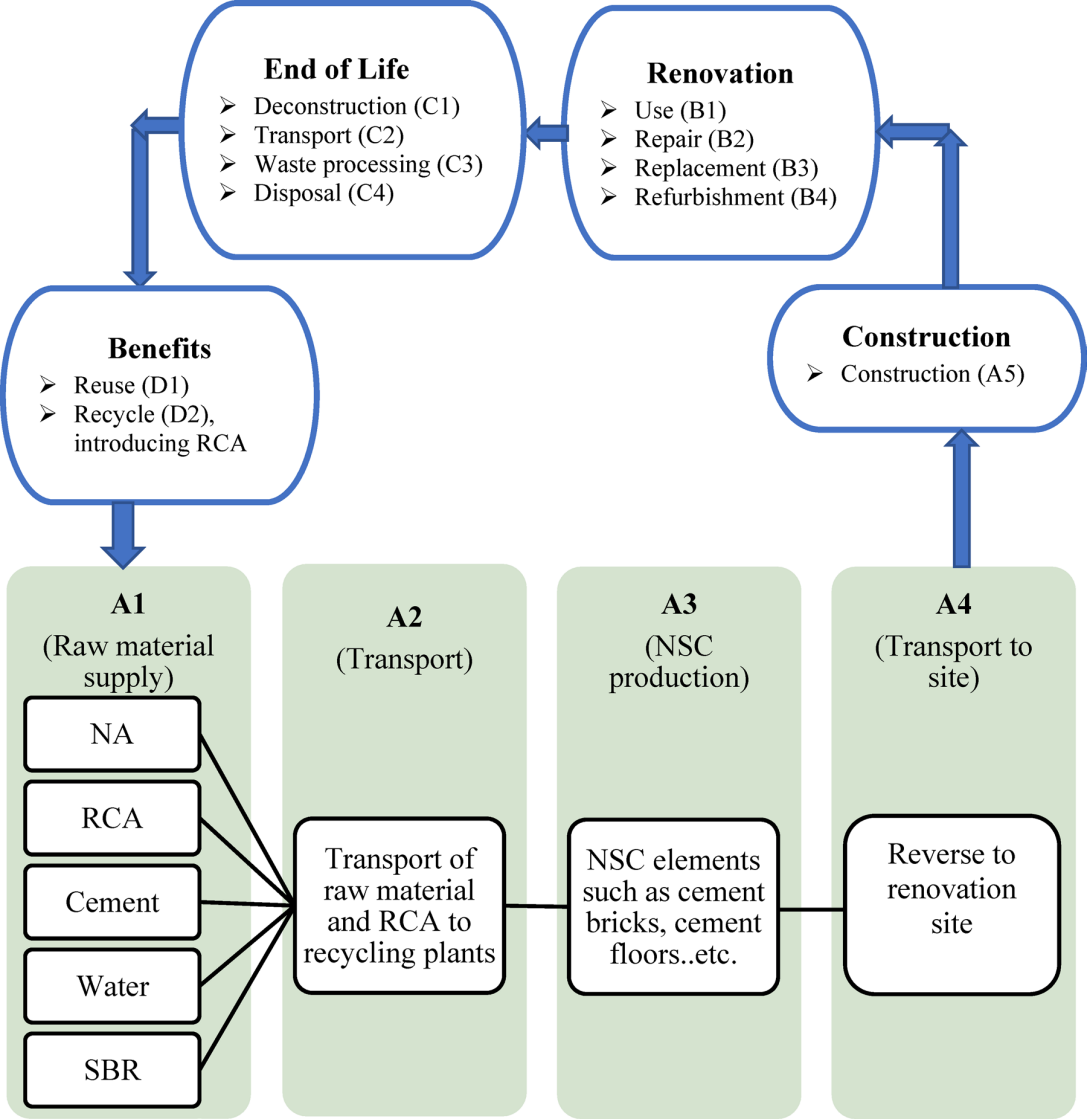
System boundary (A1-A4) for transition to circular economy in RCA recycling.

The system boundary of this study, designated as “cradle-to-site” (A1-A4) within cradle-to-cradle paradigm^55^, as illustrated in Fig. 7, envelops: the production or extraction of all essential raw materials — RCA, NA, cement, water, and SBR —for phase (A1); the transportation of those raw materials to the recycling plant (A2); the production of RAC for recycling and reusing purposes (A3); and the delivery to the construction site (A4)^56,57^.

Environmental effects assessments were conducted using SimaPro 9.6.0.1 (SimaPro 9.6.0.1, PRé Sustainability B.V., https://simapro.com/) in conjunction with the Ecoinvent 3.10 database. Ecoinvent 3.10 provides comprehensive LCI data across a wide range of products and processes, enabling the evaluation of environmental impacts from (A1-A5), (B1-B4), (C1-C4), and (D1-D2)^56^. The integration of SimaPro 9.6.0.1 with the Econivent database allows for streamlined access to data facilities, accurate and efficient LCA analysis^57^. Further researchers conducted LCA with BIM using the globally renowned International Organization for Standardization ISO 14,040 standard^58^. The subsequent step entails simulating material-related EC through the BIM-based Carbo Life Calculator, an open-source plugin for Autodesk Revit^59^.

The impact categories presented in Table 4 are evaluated using the EN 15,804 + A2 (adapted) method, providing a comprehensive description of environmental effects according to the established standards^60^. Since CO2 or EC represents the majority of GHGs (representing ~ 92%), variations in CO2 levels are generally mirrored in overall GHG trends^61^.

**Table 4:** The assessed categories using the LCA.

Impact Category	Unit
Climate change	kg CO^2^ eq
Ozone depletion	kg CFC11 eq
Ionising radiation	kBq U-235 eq
Acidification	mol H + eq
Land use	Pt
Water use	m3 depriv.
Resource use, fossils	MJ

#### Functional unit (FU)

The FU of this study is one cubic meter of concrete (1m^3^)^62^. The life cycle assessment considers inputs, including resource consumption, production processes, energy use, and transportation, alongside outputs such as waste generation, emissions, and machinery operations. To ensure comparability, the analysis is restricted to concrete mixtures characterized by similar mechanical properties and durability performance, so that each cubic meter delivers equivalent structural function. Environmental impacts are reported per 1m^3^ of concrete with comparable performance, maintaining functional equivalence.

#### Life cycle inventory (LCI)

In the LCI phase, BIM-LCA integration involves extracting material data from BIM models and associating it with the corresponding LCA data to generate LCI results^63^. During the ‘data exchange’ process, information from the BIM model is transferred into LCA tools for the calculation of LCI outcomes. Data exported from SimaPro, a widely used LCA tool, were exported (CSV) and fed into Carbo Life databases. This step ensured consistency in the mapping of materials and processes, although it introduces potential human error or variability.

To integrate LCA results within the BIM Common Data Environment (CDE), the automated material mapping within the Carbo Life Calculator plug-in was selected for its robust capabilities. This specialized tool evaluates EC using data from the SimaPro climate change category aligned file (CSV). Additionally, the tool visually maps results onto the BIM model, providing clear, component-level insights into material performance.

Data collection involved reviewing related literature and obtaining information from the database of Ecoinvent 3 LCI. For the reliability of the current study, several assumptions are established. Firstly, internal transportation effects within the CW recycling facility are not considered^64^. Additionally, it is assumed that both the recycling and batch mixing of concrete are located in local government buildings for each district, and the recycling plant is further assumed to consist of a screening facility and a fixed crusher for RCA production, with a 5:3 coarse to fine RCA ratio (Table 5). In this study, cut-off criteria are applied to record all relevant processes and materials related to CW management. This approach provides a balance between delivering a comprehensive environmental impact assessment and maintaining practicality in terms of data relevance and system complexity^65^. Furthermore, the research adopts an attributional LCA (ALCA) framework to quantify the environmental burdens of current case study in Egypt. Unlike consequential LCA (CLCA) which models dynamic impacts driven by policies or market shifts, ALCA relies on average data and defined system boundaries to capture static flows of materials, energy, and emissions, thereby offering a representative baseline for comparison^66^.

**Table 5:** LCI assumptions based ecoinvent 3.10 database and literature review.

Stage	Literature Composition	Simulated Composition	Source	Comment
A1	**FNA**	Sand [RoW], gravel and sand quarry operation | Cut-off, U	Ecoinvent 3.10 - allocation, cut-off by classification - unit	758 kg per cubic meter of concrete corresponds to the CBW0 mix, whereas 379 kg per cubic meter of concrete corresponds to the CBW1 and CBW2 mixes.
	**CNA**	Gravel, round [RoW], gravel and sand quarry operation | Cut-off, U	Ecoinvent 3.10 - allocation, cut-off by classification - unit	1263 kg per cubic meter of concrete corresponds to the CBW0 mix.
	**Cement**	Cement, Portland [RoW], cement production, Portland | Cut-off, U	Ecoinvent 3.10 - allocation, cut-off by classification - unit	250 kg per cubic meter of concrete was used in all mixes, namely CBW0, CBW1, and CBW2.
	**Water**	Tap water [RoW], market for tap water | Cut-off, U	Ecoinvent 3.10 - allocation, cut-off by classification - unit	The water-to-cement ratio for CBW0 concrete mix was 0.45, corresponding to 112.5 L per cubic meter of concrete, while for CBW1 and CBW2 mixes it was 0.6, corresponding to 150 L per cubic meter.
	**RCA**	1.74 kWh of electricity, 0.38 L of diesel, and 0.03 kg of steel are consumed per ton	^67^	The recycling process was defined in SimaPro based on the energy needed for the process obtained from literature. Moreover, 1263 kg CRCA and 379 kg FRCA per cubic meter of concrete were used in the CBW1 and CBW2 mixes.
	**SBR**	Synthetic rubber [GLO], market for synthetic rubber | Cut-off, U	Ecoinvent 3.10 -allocation, cut-off by classification - unit	The SBR-to-water ratio for CBW1 concrete mix was 1:25, corresponding to 6 kg per cubic meter of concrete, while for CBW2 mix it was 1:50, corresponding to 3 kg per cubic meter.
A2		Transport, freight, lorry 16–32 metric ton, EURO5 [RoW], transport, freight, lorry 16–32 metric ton, EURO5 | Cut-off, U	Ecoinvent 3.10 - allocation, cut-off by classification - unit	120.0 km for each FNA, CNA, and cement. On the other hand, the study assumes an average distance of 5.0 km (CBW1 and CBW2) from the CW source to the decentralized recycling plant. Distance to centralized recycling plant around 20.0 km (CBW0)
A3	Concrete plant (1 m^3^ manufacturing)	The electricity needed is 2.0 kWh. Diesel fuel consumption, including the mixing process, is assumed as 12.65 L, considering a diesel density of 0.84 kg/L	^68^	The mixing process was defined in SimaPro based on the energy needed for the process obtained from literature.
A4	Non- structural concrete elements	Transport, freight, lorry 3.5–7.5 metric ton, EURO3 [RoW], transport, freight, lorry 3.5–7.5 metric ton, EURO3 | Cut-off, U	Ecoinvent 3.10 - allocation, cut-off by classification - unit	The study assumes the reverse logistics (RL) distance from the recycling plant to sites is 5.0 km.

## Results and discussions

### Experimental observations

#### NA and RCA properties

**Fig. 8:**
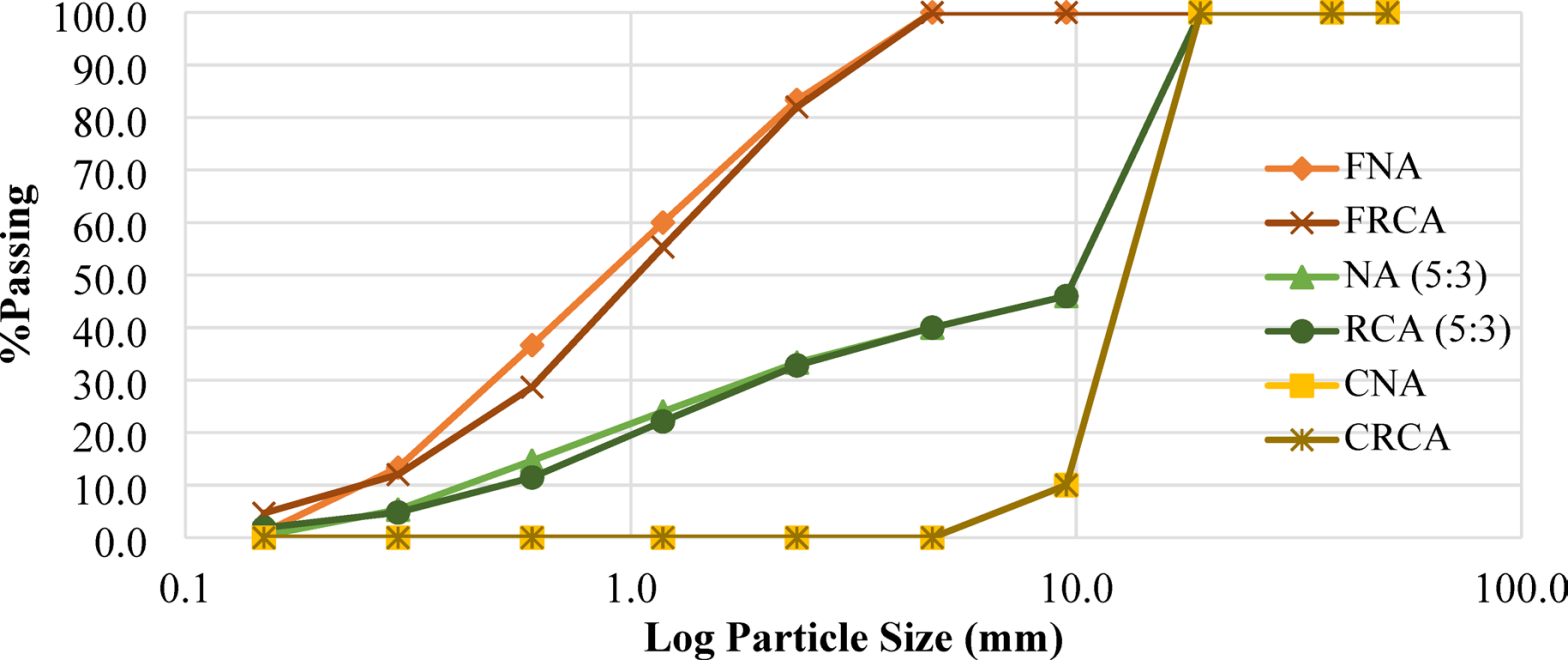
Aggregates’ Particle size distribution.

A sieve analysis was performed according to ASTM C136/C136M to determine the PSD of the aggregates^45^. The PSD curves (Fig. 8) indicate that the grading complies with the limits recommended for concrete production in ASTM C33/C33M^69^, with a maximum particle size of 9.50 mm. The use of RCA in concrete production substantially affects both material properties and mix design due to the presence of adhered mortar and other impurities. These factors alter the chemical composition of RCA, leading to changes in density, specific gravity, and water absorption^70^. Additionally, the crushing process imparts an angular shape to the aggregate^71^. A comparison with ASTM specification limits revealed that both CRCA and FRCA generally exhibit lower specific gravity, reduced bulk density, and higher absorption compared to NA^72^. In the present study, the bulk densities of CNA and CRCA were comparable and within standard limits as shown in Table 6, whereas FRCA showed a bulk density approximately 21% lower than that of FNA.

**Table 6:** Aggregate physical properties for NA and RCA.

Property	Coarse aggregates		Fine aggregates		Range	Standard test
	CRCA	CNA	FRCA	FNA		
Bulk density (kg/m^3^)	1315	1351	1275	1610	1200–1750	ASTM29^75^
Specific gravity	2.53	2.83	2.36	2.87	2.40–2.90	ASTM 127/128^76^
Water absorption (%)	8.19%	2.72%	11.86%	2.68%	0.2%−4%	ASTM 127/128^77^^[,78^
FM	-	-	3.17	3.06	2.3–3.1	ASTM C33/C33M^79^
Clay and silt content (%)	-	-	4.38%	0.94%	Not more than 5%	ASTM C142/C142M^80^

In literature, Wagih et al. reported that CRCA exhibited a specific gravity of 2.37 and a bulk density of 1.34 t/m^3^, which are comparable to the values obtained in the present study. Moreover, they recorded an abrasion index of 35 and an impact value of 23, both of which are within the acceptable limits specified by ASTM C535^8^. These findings support the suitability of the RCA used in this work, even though aggregate-level mechanical tests were not directly performed.

#### Fresh properties of RAC (Workability indices)

**Fig. 9:**
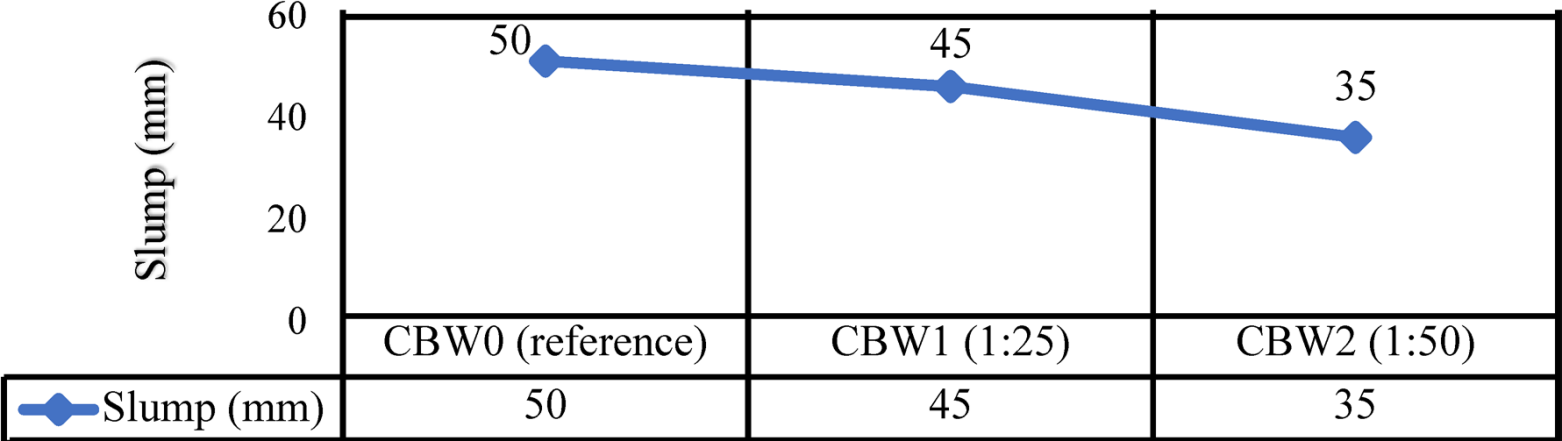
Slump values versus the percentage of SBR in recycled concrete mixes.

The workability of concrete refers to how easily it can be prepared, applied, compacted, and finished while keeping its homogeneity and causing minimal disruptions^79^. Slump and slump flow are important measures for fresh concrete workability assessment^80^. Moreover, with the increase in RCA rate of replacement, slump and flowability decreased^81^. The ASTM C143, which describes medium and low concrete having a slump within a range of 25 mm to 50 mm, served as the basis for the slump test^82^. Figure 9 displays the slump test values for the prepared mixes. The slump values of CBW1 and CBW2 decreased by 10.0% and 30.0% in comparison to CBW0, respectively. Thus, it indicates that increasing the amount of SBR along with RCA enhances the workability of concrete.

#### Mechanical properties of RAC

**Fig. 10:**
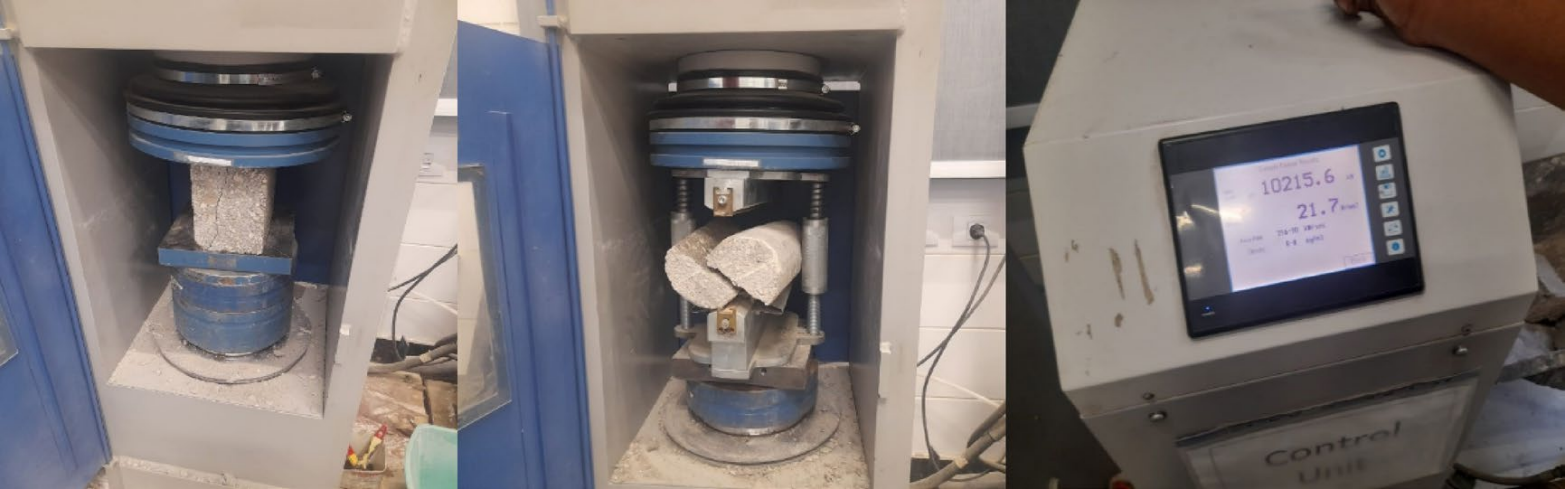
Compressive strength and indirect tensile strength tests.

**Fig. 11:**
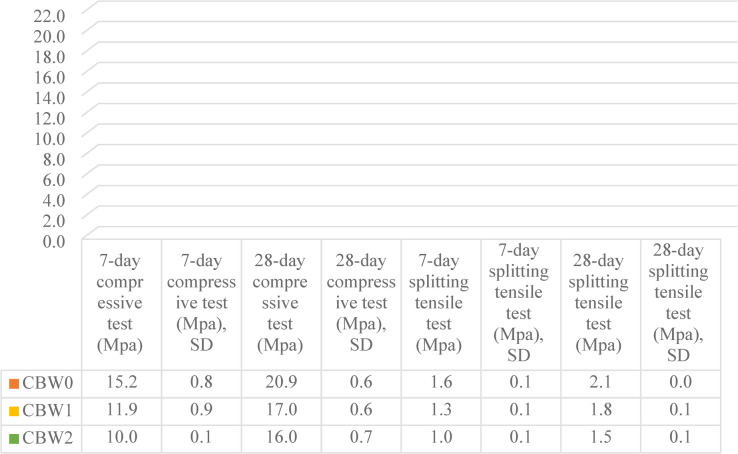
The average compressive and splitting tensile strength of mixes.

Compressive strength tests at 7 and 28 days were conducted according to the ASTM C39 guidelines, Fig. 10^83^. The average values for each mix, obtained by crushing under the compressive load, are shown in Fig. 11. They illustrate the values of compressive strength for mixtures CBW1 and CBW2 are 17.0 and 16.0 MPa, which are applicable for most NSC applications. The European code establishes that non-structural concrete must achieve a minimum characteristic compressive strength of 15 MPa and contain at least of 150 kg/m^3^ cement^84^. In line with this requirement, a compressive strength of around 15 MPa in concrete incorporating mixed recycled aggregate is considered adequate for many NSC applications^84^. Both CBW1 and CBW2 satisfy this performance threshold. While higher RCA content typically reduces strength, the addition of SBR elevated the bond between old and new mortar, contributing to improved mechanical performance^85^. In its liquid form, it reduces the mixing water demand and lowers the water-to-cement ratio, thereby boosting durability and strength without compromising workability^86^. SBR latex also augments the freeze-thaw resistance of concrete and helps reduce shrinkage-induced cracking^87^. Consequently, CBW, which contained a higher SBR dosage, manifested greater compressive strength than CBW2, which had a lower SBR content. Concrete’s splitting tensile strength generally corresponds to approximately 8–12% of its compressive strength^88^. Previous studies have reported that substituting NA with RCA may influence tensile performance^89^. Figure 11 presents the tensile strength results obtained under indirect tensile loading by ASTM C496^90^. Each value represents the mean of three replicate specimens per mix. Furthermore, the low SD values indicate that the mixtures exhibit a high degree of homogeneity.

### Environmental results

#### LCIA of concrete mix and aggregate

**Fig. 12:**
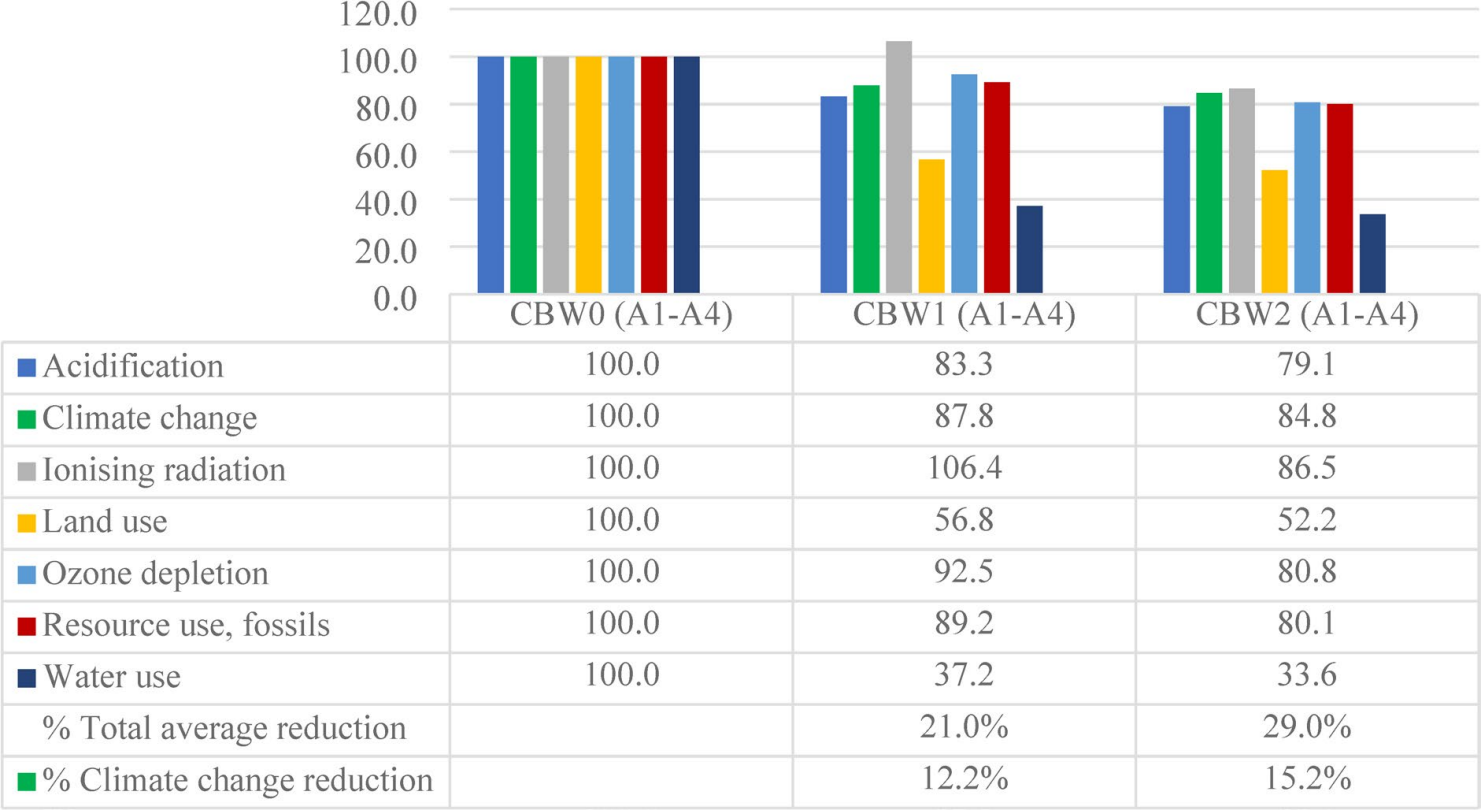
Weighting the total environmental impacts of 1m^3^ concrete production (cradle to site) for “CBW1”, “CBW2” and “CBW0”.

LCIA provides a quantitative evaluation of the environmental footprint derived from the LCI results^91^. The life cycle impact assessment (LCIA) outcomes for A1-A4 phases are presented in Table 7; Fig. 12. The environmental burdens of the three concrete mixes were assessed using the EN 15,804 + A2 (adapted) method for each impact category. Additionally, the bar chart illustrates the comparative contributions of environmental effects for each mix.

**Table 7:** Life cycle impact assessment – A1 to A4 phases.

Impact category	Unit	CBW0	CBW1	CBW2
Acidification	mol H + eq	1.04	0.86	0.82
Climate change	kg CO2 eq	327.30	287.44	277.56
Ionising radiation	kBq U-235 eq	1.32	1.40	1.14
Land use	Pt	988.06	560.97	515.65
Ozone depletion	kg CFC11 eq	2.42E-06	2.23E-06	1.95E-06
Resource use, fossils	MJ	2747.99	2450.53	2202.24
Water use	m3 depriv.	143.84	53.44	48.28

The results of this study highlight substantial reductions in environmental burdens when comparing the recycled concrete mixes to the reference mix (CBW0). Mixes CBW1 and CBW2 achieved average savings of 21% and 29%, respectively. Among the impact categories, water use was the most strongly affected by the partial replacement of NA with RCA and SBR, with declines of 62.8%, and 66.4% for CBW1 and CBW2, respectively.

Regarding climate change impacts per cubic meter, CBW2 demonstrated a notable 15.2% decreases compared to CC. These findings are consistent with prior studies reporting that substituting NA with RCA can reduce total energy consumption^92^. Nevertheless, not all research has observed similarly improvements; for example, a study of concrete block production with RCA reported minimal benefits across most impact categories^93^.

**Fig. 13:**
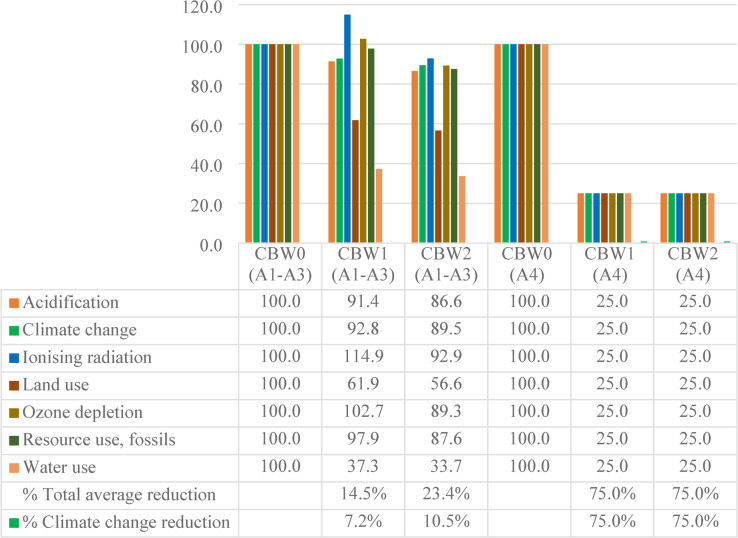
Weighting the environmental effects for phases (A1-A3), and A4 of 1m^3^ concrete production for “CBW1”, “CBW2” and “CBW0”.

Moreover, Fig. 13 illustrates GHGs for A1-A3 and A4 life cycle phases for each mix. The analysis reveals variable reductions across these stages, with particularly pronounced decreases during A4. Specifically, CBW2 exhibited mitigations of 23.4% in A1-A3, and 75% in A4 compared to the reference mix. The primary contributor to this improvement is the reverse transportation savings achieved through the use of decentralized recycling facilities. Regarding the EC, CBW2 exhibited a moderate 10.5% reduction compared to CBW0 in phases (A1-A3). On the other hand, a notable decrease in A4 phase with percentage 75%. These observations align with earlier research, highlighting the substantial contribution of transport distance on the ecological benefits associated with the use of recycled aggregates^94^.

**Fig. 14:**
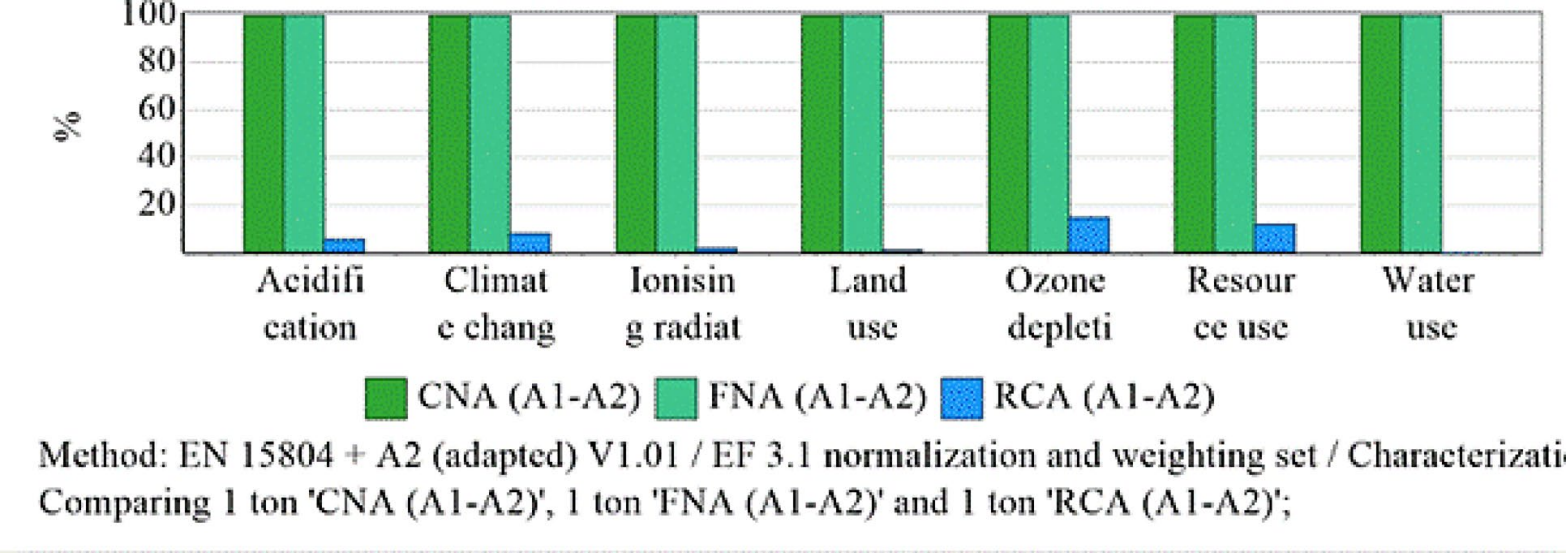
Characteristics the environmental effects for phases A1-A2 of one ton aggregate.

Figure 14 illustrates the environmental effects for A1-A2 phases of 1 ton of aggregate. This gate-to-gate comparison isolates the impact of CRCA and FRCA relative to CNA and FNA, showing that GHGs and EC for RCA are reduced by 95% compared to NA. These results highlight the significant environmental advantages of using RCA and underscore the need for carful, context-specific evaluation in sustainable concrete production.

#### Automation BIM-LCA for NSC elements

While the FU remains defined at the material level (1m^3^ of RAC/NSC), these results were upscaled through BIM-based element modelling to illustrate their relevance implications at the building element scale. Table 8 presents the EC of three concrete mix scenarios for ground concrete floor elements (CBW0, CBW1, and CBW2), calculated with a BIM-based automation tool. The EC values were 33.3 tons for CBW0, 29.3 tons for CBW1, and 28.3 tons for CBW2, covering life cycle stages A1-A3 and A4. Compared to the baseline (CBW0), CBW2 achieved 15.2% reduction in EC, making it the most favorable option for low-carbon NSC elements. These findings also demonstrate that BIM-enabled automation improves both the speed and accuracy of EC assessment relative to traditional methods^95^, thereby supporting reliable early-stage evaluations and more sustainable material and design decisions.

**Table 8:** EC contributions by material and element with mapping results for the CBW0, CBW1, and CBW2 Scenarios.

Category	Mix	Total Volume (m^3^)	Density (kg/m³)	Mass (kg)	A1-A3 (tCO2	A4 (tCO2	EC (tCO2
Floors	CBW0	101.9	2383.5	242791.2	30,909	2,412	33,322
	CBW1				28,670	608	29,280
	CBW2				27,670	608	28,273
	% Reduction of CBW1 vs. CBW0				7.2%	75%	12.1%
	% Reduction of CBW2 vs. CBW0				10.5%	75%	15.2%

In conclusion, the quantitative assessment of environmental performance conducted in this study provides a robust framework for evaluating alternative material options and related efficiencies using BIM, thereby supporting informed decision-making in sustainable construction.

## Conclusions

This study investigated the physical, fresh, and mechanical properties of RAC designed for NSC applications with a target compressive strength of 20 MPa. Furthermore, the study assessed the carbon footprint associated with recycling scenarios using a semi-automated BIM-LCA framework. The key conclusions are summarized as follows:

- **Superior performance of CBW2**: CBW2 achieved the lowest environmental burdens, with a 15.2% reduction in EC compared to the baseline CBW0, proving the effectiveness of maximizing RCA.
- **Environmental advantage of RCA**: On gate-to-gate basis (A1-A2), RCA showed over 95% lower GHGs and EC impacts compared to NA, confirming their role as a sustainable material alternative.
- **Mechanical performance**: CBW2 (100% coarse RCA + 50% fine RCA, SBR/W = 1:50) and 250 kg/m^3^ cement content produced concrete up to 16 MPa compressive strength, which is feasible for most NSC applications.
- **Transport and logistics benefits via decentralization**: The adoption of decentralized recycling plants greatly reduced transportation distances in phases A2 and A4, leading to up to 75% lower GHGs. This approach not only cuts emissions but also support local CE practices by keeping resources within urban construction loops.
- **BIM-LCA automation advantages**: The BIM-integrated Carbo Life Calculator enabled fast, accurate, and early-stage EC assessments, outperforming traditional manual methods. This efficiency supports more responsive design iterations and informed low-carbon material selection.
- **Strategic pathway for sustainable design**: The combination of CBW2 mix design and decentralized recycling plants, integrated with BIM-LCA automation, provides a scalable and practical solution to reduce EC in NSC elements and aligns with sustainable goals.

## Limitations and recommendations for future research

### Limitations of the study

This study contributes to the advancement of BIM-LCA integration for decentralized CBW recycling in renovation projects, demonstrating its potential to support CE strategies in the CDW sector. However, several limitations should be acknowledged:

- **Technological and organizational limitations**: Although BIM-LCA integration was successfully applied in the case study, the technological complexity and financial burden of BIM adoption may constrain its widespread use, especially among small and medium-sized enterprises. The scalability of the approach and its integration into firms with limited resources and digital expertise remain uncertain.
- **Long-term performance and material durability**: The analysis primarily captured the immediate environmental benefits of using RCA and additives such as SBR latex in concrete. Long-term durability under varying climate conditions and extended service life were not assessed. Regarding fresh properties, evaluation in this study was limited to the slump test due to resource and scope constraints. Nevertheless, we fully recognize the importance of more comprehensive workability assessments (e.g., slump flow per En 12350–5, V-funnel, L-box, and rheological measurements).
- **Contextual feasibility of decentralized recycling plants**: While the study illustrates potential benefits of localized recycling facilities, their real-world deployment may be hindered by regulatory requirements, logistical challenges, and economic constraints. These factors require further examination before decentralized strategies can be scaled across diverse urban contexts.

### Recommendations for future research

Building on these limitations, future research should address the following directions:

- **Broader geographic and contextual applications**: Extend case studies across diverse climate zones, urban vs. rural settings, and regions with different regulatory and data availability conditions. This would provide a more comprehensive understanding of how BIM-LCA frameworks can be adapted to heterogeneous construction environments. Furthermore, the inclusion of a sensitivity analysis for (e.g. variations in SBR/W ratios and transportation distances) are recommended, as it would substantially strengthen the robustness and reliability of environmental assessment.
- **Technological innovation and accessibility**: Develop simplified, cost-effective, or open-source BIM solutions that are accessible to smaller firms. Enhancing affordability and usability would promote wider adoption of CE-oriented digital workflows.
- **Integrated environmental**,** economic**,** and social assessments**: Expand the analytical scope beyond environmental performance to include economic feasibility (e.g., life cycle cost analysis) and social dimensions such as employment generation, local economic impacts, and community benefits from reduced landfill use. Such integration would provide a more holistic basis for decision-making.

By critically addressing these limitations and advancing the above research directions, future studies can strengthen the application of BIM-LCA integration for decentralized CBW recycling in renovation projects. This will not only reduce the environmental footprint of CW but also foster resource efficiency, economic, viability, and social value in line with CE objectives.

## Supplementary Information

Below is the link to the electronic supplementary material.

Supplementary Material 1

Supplementary Material 2

## Data Availability

All data (data collected from industry- analysis -output after analysis) generated or analyzed during this study are included in this published article and its supplementary information files.

## Abbreviations

CDW: Construction and demolition waste
CW: Concrete waste
CBW: Cement-based waste
CE: Circular economy
GHGs: Greenhouse gas emissions
CO_2_ (EC): Carbon dioxide (Embodied carbon), or climate change
RCA: Recycled concrete aggregates
CRCA, FRCA: Coarse and fine recycled concrete aggregates, respectively
NA: Natural aggregates
CNA, FNA: Coarse and fine natural aggregates, respectively
SBR (ADD 65): Styrene butadiene rubber (Addi-bond 65)
W: Water
CC: Conventional concrete
RAC: Recycled aggregate concrete
ACI: American concrete institute
ASTM: American society for testing and materials
PSD: Particle size distribution
FM: Fineness modulus
SD: Standard deviation
NSC: Non-structural concrete
CAD: Computer-aided drafting
BIM: Building information modelling
MPs: Material passports
LOD: Level of details / Level of development
LCA: Life cycle assessment
LCI: Life cycle inventory
LCIA: Life cycle impact assessment
A1: Raw material supply
A2: Transportation
A3: Concrete production
A4: Reverse transport to the site
